# Dose-response relationships of intestinal organs and excessive mucus discharge after gynaecological radiotherapy

**DOI:** 10.1371/journal.pone.0250004

**Published:** 2021-04-16

**Authors:** Eleftheria Alevronta, Viktor Skokic, Gail Dunberger, Cecilia Bull, Karin Bergmark, Rebecka Jörnsten, Gunnar Steineck

**Affiliations:** 1 Division of Clinical Cancer Epidemiology, Department of Oncology, Institute of Clinical Sciences, Sahlgrenska Academy at the University of Gothenburg, Gothenburg, Sweden; 2 Department of Health Care Sciences, Ersta Sköndal Bräcke University College, Stockholm, Sweden; 3 Chalmers University of Technology, Gothenburg, Sweden; 4 Division of Clinical Cancer Epidemiology, Department of Oncology and Pathology, Karolinska Institutet, Stockholm, Sweden; St. Vincent Medical Center, UNITED STATES

## Abstract

**Background:**

The study aims to determine possible dose-volume response relationships between the rectum, sigmoid colon and small intestine and the ‘excessive mucus discharge’ syndrome after pelvic radiotherapy for gynaecological cancer.

**Methods and materials:**

From a larger cohort, 98 gynaecological cancer survivors were included in this study. These survivors, who were followed for 2 to 14 years, received external beam radiation therapy but not brachytherapy and not did not have stoma. Thirteen of the 98 developed excessive mucus discharge syndrome. Three self-assessed symptoms were weighted together to produce a score interpreted as ‘excessive mucus discharge’ syndrome based on the factor loadings from factor analysis. The dose-volume histograms (DVHs) for rectum, sigmoid colon, small intestine for each survivor were exported from the treatment planning systems. The dose-volume response relationships for excessive mucus discharge and each organ at risk were estimated by fitting the data to the Probit, RS, LKB and gEUD models.

**Results:**

The small intestine was found to have steep dose-response curves, having estimated dose-response parameters: *γ*_*50*_: 1.28, 1.23, 1.32, *D*_*50*_: 61.6, 63.1, 60.2 for Probit, RS and LKB respectively. The sigmoid colon (AUC: 0.68) and the small intestine (AUC: 0.65) had the highest AUC values. For the small intestine, the DVHs for survivors with and without excessive mucus discharge were well separated for low to intermediate doses; this was not true for the sigmoid colon. Based on all results, we interpret the results for the small intestine to reflect a relevant link.

**Conclusion:**

An association was found between the mean dose to the small intestine and the occurrence of ‘excessive mucus discharge’. When trying to reduce and even eliminate the incidence of ‘excessive mucus discharge’, it would be useful and important to separately delineate the small intestine and implement the dose-response estimations reported in the study.

## Introduction

Few, if any, survivors who have undergone radiotherapy for a pelvic tumor return to the intestinal health they had before treatment. Pelvic-radiation disease contracted during radiotherapy may consist of as many as 30 different late adverse intestinal effects [1] that compromise the survivor’s daily life. One such late adverse effect is excessive mucus discharge or ‘mucus syndrome’, which is a patient-reported outcome. We do not know the extent to which ionizing radiation delivered to the small intestine, sigmoid colon, or rectum may be responsible for inducing this specific late adverse effect.

The small intestine protects its epithelium from stressors by increasing the production of mucus. It appears possible that ionizing radiation reaching the small intestine may act as a mucus-producing stressor [2]. During normal conditions, mucus from the small intestine reaching the large intestine is degraded to short-chain fatty acids by the action of bacteria in the microbiota in the large intestine. These short-chain fatty acids are absorbed in the intestinal wall, leading to, among other things, restoration of the energy that was used during the production of mucus [3]. It is also possible that ionizing radiation to the sigmoid colon or rectum compromises the degradation of mucus by inducing dysbiosis. Thus, we have rationales for investigating the extent to which excessive mucus discharge is related to the ionizing radiation delivered to both the small and large intestine.

Our study investigated and quantified the dose-volume response relationships of the small intestine, sigmoid colon and rectum to the ‘mucus syndrome’. The ultimate aim was to predict the occurrence of the mucus syndrome as a result of the radiation dose to the organs at risk (OAR). For that purpose, we employed various statistical analyses and normal tissue complication (NTCP) models, using as an outcome a score, measuring the degree of excessive mucus discharge.

## Methods and materials

From a larger group of 650 cancer survivors who had been treated with radiation therapy for gynecological cancer, 98 were selected for this study. From the 650 individuals 20 were excluded due to having a bowel stoma. Among the 630 remaining 7 were excluded due to having more than 30% missing values across the 28 studied symptoms. Of the 623, 98 received no brachytherapy and a DVH could be extracted from the treatment planning system. Thirteen of the 98 had developed ‘mucus syndrome’. Patients clinical information were retrieved from the medical records. The OARs that were studied were the rectum, the sigmoid colon and the small intestine. The OARs were delineated and the dose-volume histograms were exported for each survivor from the treatment planning systems. The survivors included in the study had received combinations of external radiation therapy (EBRT), surgery, and chemotherapy (Table 1) at Karolinska University Hospital, Stockholm or Sahlgrenska University Hospital, Gothenburg during 1991–2003. The study was approved by the Regional Ethical Review Board (2005/1424-31/4), Stockholm, Sweden. The survivors were informed about the study and agreed to participate. According to the Ethical Review Board, completing the questionnaire and sending it by post, is considered to be a written consent of participation. The main data collection was carried out between January to October 2006. The extraction of the dose data was carried out between 2006 and 2007. For the analysis, the data were accessed fully anonymized.

**Table 1:** Demographic, clinical characteristics and treatment characteristics of survivors with or without ‘excessive mucus discharge’ after pelvic radiation therapy.

	Survivors with the syndrome	Survivors without the syndrome	*p*-value
	*N = 13*	*N = 85*	
***Age*** *(SD)*			
Mean age, years	61.9 (10.2)	65.9 (7.5)	0.29
***Smoking-*** n/N (%)			0.39
Current smoker		24/84 (29)	
Former smoker	7/12 (58)	34/84 (40)	
Never smoker	5/12 (42)	26/84 (31)	
***Deliveries-*** *n/N (%)*			0.063
0	9/12 (75)	70/84 (83)	
1	2/12 (17)	12/84 (14)	
≥2	1/12 (2)	2/84 (2)	
***Pelvic floor injury-*** *n/N (%)*			
Perineum injury	2/12 (17)	24/85 (28)	0.51
Anal sphincter injury	0/24 (0)	4/74 (5)	0.57
***Co-morbidities-*** *n/N (%)*			
Rheumatism	2/12 (17)	8/85 (9)	0.61
Diabetes mellitus	10/12 (4)	7/86 (11)	0.30
Hypertension	6/12 (50)	24/85 (27)	0.17
Hemorrhoids, treatment for	1/12 (8)	5/86 (7)	0.55
Lactose intolerance	0/12 (0)	4/86 (5)	1.00
**Diagnoses*-*** *n/N (%)*			0.65
Endometrial cancer	0/12 (0)	9/86 (10)	
Cervical cancer	3/24 (12)	15/86 (17)	
Ovarian cancer	5/12 (42)	28/86 (33)	
Vulvar cancer	1/12 (8)	5/86 (6)	
Vaginal cancer	0/12 (0)	3/86 (3)	
Sarcoma	1/12 (8)	17/86 (20)	
Fallopian tube cancer	3/12 (25)	9/86 (10)	
**Treatment characteristics**			
***Treatment modalities-*** *n/N (%)*			0.20
EBRT+ Surgery	3/12 (25)	39/86 (45)	
EBRT + Surgery + Chemotherapy	7/12 (58)	0/86 (47)	
EBRT+ Chemotherapy	1/12 (8)	6/86 (7)	
EBRT only	1/12 (8)	1/86 (1)	
***EBRT doses*** *(SD)*			
Total mean dose Gy	45.2 (9.8)	45 (11.2)	0.71
***Field technique-*** *n/N (%)*			0.21
Two opposing fields	8/12 (67)	40/85 (47)	
Four-field box	4/12 (33)	45/85 (53)	
***Target area-*** *n/N (%)*			0.32
Pelvic field	3/12 (25)	39/86 (45)	
Lower Abdominal field	8/12 (67)	38/86 (44)	
Abdominal field	1/12 (8)	2/86 (2)	
Pelvic field + paraaortic lymph nodes	0/12 (0)	3/86 (3)	
Pelvic or vulvar field + inguinal lymph nodes	0/12 (0)	4/86 (5)	
***Mean time since EBRT*, *months (SD) (SD)(SD)***			
	7.6 (3.3)	8.8 (4)	0.34

As described in a previous study by our group [4], a validated postal questionnaire was constructed consisting of 351 questions concerned with physical symptoms, quality of life, social functioning, and demographics [5]. 28 questions dealing with intestinal-health symptoms were analysed using exploratory factor analysis based on the Spearman correlation [6]. Parallel analysis [7] as well as a bootstrap version of Kaiser’s rule [8, 9] suggested that six factors best described the structure of the data. After applying the Variamax rotation to the fitted factor analysis model, the Variable Cutoff Method, described in the appendix to [6], was applied to enhance the interpretability of the factor loading structure by making it sparse, i.e. setting some factor loadings to zero. Based on this sparse factor loading structure five of the factors were interpreted as radiation induced long term intestinal syndromes. Factor scores, interpreted as syndrome intensities, were further calculated based on this sparse version of the factor loading structure. The analyses were performed in R using in particular the fa() from the psych package to fit the exploratory factor analysis model to the data.

The 98 survivors had received EBRT according to International Commission on Radiation Units and Measurements (ICRU 1993) [10]. The EBRT plan was made based on Computer Tomography (CT) scans; the thickness of the slices was 5–20 mm. The treatment planning system used in Stockholm was the TMS (Nucletron, Veenendaal, the Netherlands), while in Gothenburg the Cadplan and Eclipse systems (Varian Medical Systems, Palo Alto, CA, USA) were used. Patients were treated when in a supine position using linear accelerators with energy ranging from 6 to 50 MV. The fractionation schedule was 1.6, 1.8, and 2.0 Gy per fraction.

The survivors were treated for endometrial cancer, cervical cancer, ovarian cancer, vaginal, sarcoma, and fallopian tube cancer. For endometrial cancer the prescribed doses were 40–46 Gy, and for uterine sarcomas 50 Gy. For cervical cancer, the total prescribed dose was 55.70 Gy, administered in two phases. The first phase was administered using similar techniques to the above diagnoses; in the second phase, a boost was used targeted on a smaller volume. For ovarian and fallopian tube cancers 20 Gy was given to the whole abdomen and pelvis, and an additional 20 Gy to a reduced volume delimited by lowering the cranial margin.

The rectum, the sigmoid colon and the small intestine were contoured in Stockholm and Gothenborg using the same written guidelines. Our method has been thoroughly described in our previous publication [11]. The rectum was delineated by including the filling, and as delineated it extended from the anal verge to the rectosigmoid junction. The beginning of the sigmoid colon was contoured where the rectum deviates from its midposition and extended to where it turns cranially in the left part of the abdomen connecting to the colon descendens. Finally, the small intestine was delineated as the volume between the pelvic cavity and the caudal part of the sacroiliac joints [11].

For the rectum and sigmoid colon, we produced the cumulative DVHs for each patient. For the small intestine, the absolute volume was used for the DVHs because we delineated only a part of the organ. To be able to compare the DVHs for survivors with and without the syndromes of each cancer survivor, we calculated the mean cumulative DVH for each group. We corrected the physical dose-distribution to 2 Gy/fraction using the Biological Effective Dose (BED) [12] to account for differences in the fractionation schedule. In order to do basic calculations and radiobiological calculations, we used BML software based on the optimisation software Nonlinear Programming, Systems Optimization Laboratory NPSOL [13].

We calculated the dose-volume response relationships of the urgency syndrome and the OARs. We fitted the data to the Probit model [14], which is a dose-response model describing the sigmoidal shape of the dose-response curve and based on the cumulative dose-distribution.

$$P(D)=\frac{1}{2}(1-Erf[\gamma_{50}\sqrt{\pi }(1-\frac{D}{D_{50}})]).$$ (1)

The Probit model is conveniently parametrised with *D*_*50*_, the dose corresponding to a 50% response probability after uniform irradiation of the reference volume and *γ*_*50*_, which is the maximum normalised dose-response gradient at 50% response probability.

We also fitted the data to the Relative Seriality (RS) [15] and the Lyman Kutcher Burman (LKB) [16] models, which are dose-volume response models that also account for the volume effect. The response probability with the RS which is given by:

P(D→,V→)=[1−∏i=1M(1−P(Di,Vref)s)ViVref]1/s, (2)

where *V*_*i*_ is the irradiated subvolume of an organ compared to the reference volume, *V*_*ref*_ and *s* is the RS parameter that characterizes the structural organization of the Functional Subunits (FSUs) in the organ and account for the volume effect. *M* is the total number of voxels in the organ and *P*(*D*_*i*_, *V*_*ref*_) is the probability of response of the organ having reference volume *V*_*ref*_ and being uniformly irradiated to dose *D*_*i*_. The response probability P(D) of the OARs and the mucus syndrome used in the RS model, were calculated using the Probit model.

In the Lyman model, the probability of observing a specified complication after irradiation to dose *D* of subvolume *V*, expressed as a proportion of the whole organ or other reference volume, is modeled using the Probit function:

$$P(D,V)=\frac{1}{\sqrt{2\pi }}\int_{-\infty }^{t}e^{-t^{2}/2}dt$$ (3)

where

$$t(D,V)=\frac{D-D_{50}V^{n}}{mD_{50}{/V}^{n}},$$ (4)

where *n* is the volume parameter and *m* is inversely related to the slope of the dose–response curve or the normalized dose response gradient *γ*_*50*_ ($m=\frac{1}{\gamma_{50}\sqrt{\pi }}$). We combined the Lyman model with the generalized Equivalent Uniform Dose (gEUD) model [17, 18] but Lyman could be combined with any DVH reduction model. The gEUD is used to summarise the whole dose-distribution in a volume of interest to a single value and is given by the following equation:

$$gEUD={\left(\frac{1}{N}\sum_{i=1}^{N}D_{i}^{\alpha }\right)}^{n},$$ (5)

The *n* parameter is inversely related to the *α* parameter of g EUD model $\left(\alpha =\frac{1}{n}\right)$.

The model parameters were estimated using the maximum likelihood method setting *α/β* ratio equal to 1. The *α/β* ratio is defined as a dose *α/β*, where the linear and quadratic components of cell killing of the linear quadratic equation are equal. To evaluate the goodness of fit of each model, the Log Likelihood (LL) and the Akaike Information Criterion (AIC) were used. In the AIC method, the LL is adjusted for the number of parameters of each model in order to balance model fit and model complexity. To evaluate the uncertainty of the model parameter values, the 68% confidence intervals (CI) were calculated. The Area under the ROC curve (AUC) was used to describe the probability that a model will correctly separate the survivors with the syndrome from survivors without the syndrome. When comparing distributions of categorical variables p values were calculated using Fishers exact test. In cases of continuous variables the Mann-Whitney test was used. Moreover, the Pearson correlation coefficient r was calculated, to investigate the volume parameters.

## Results

**Fig 1:**
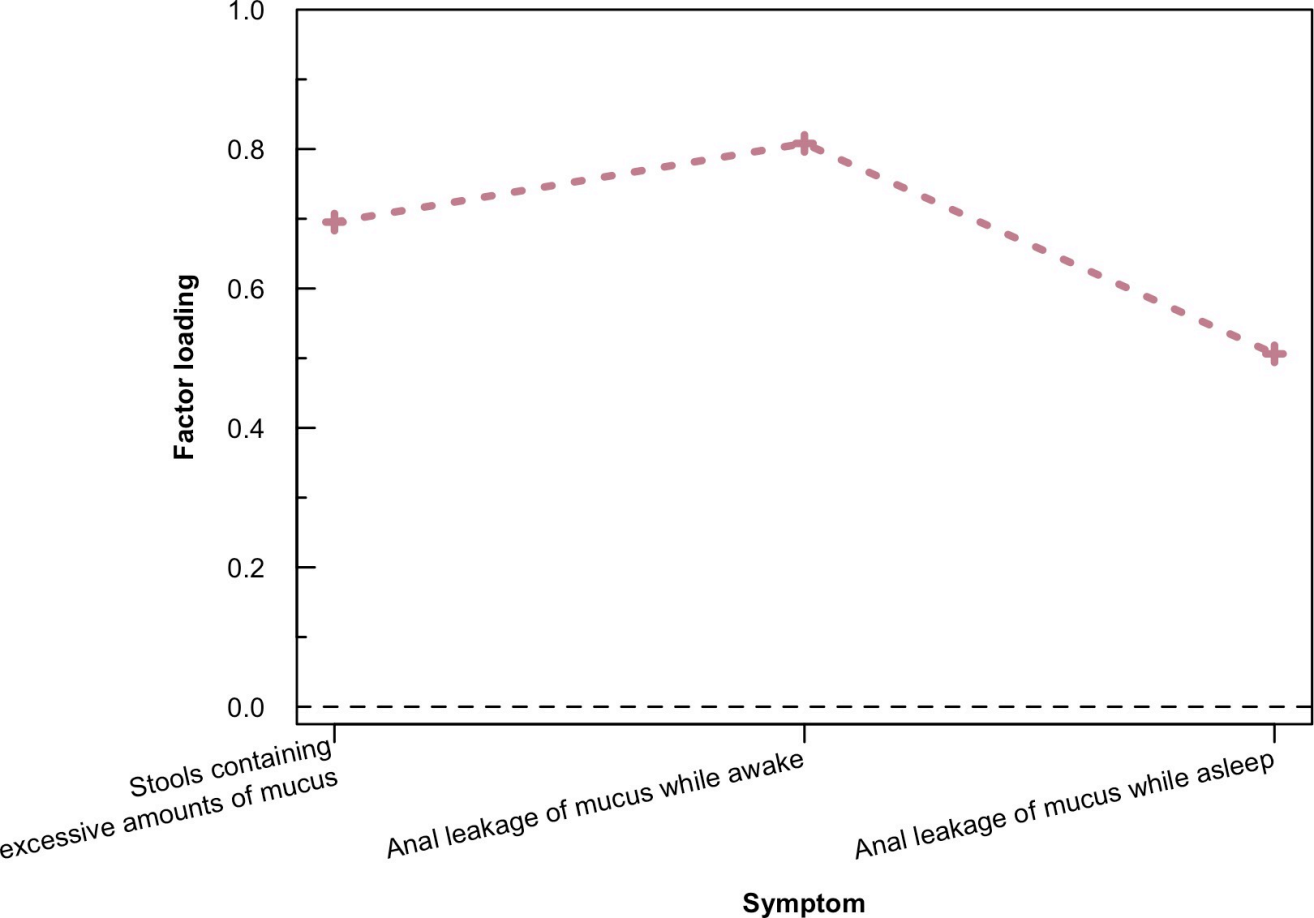
Factor loadings associated with ‘excessive mucus discharge’. The crosses illustrate the factor loadings corresponding to the three symptoms associated to ‘excessive mucus discharge’.

Table 1 summarises the demographics and treatment characteristics of the 98 cancer survivors with and without ‘mucus syndrome’ included in the study. There was no significant association found between the occurrence of ‘mucus syndrome’ and the studied variables. Fig 1 illustrates the factor loadings for the three symptoms which are associated and constitute the factor ‘mucus syndrome’. Of these, ‘anal leakage of mucus while awake’ had the highest loadings.

**Fig 2:**
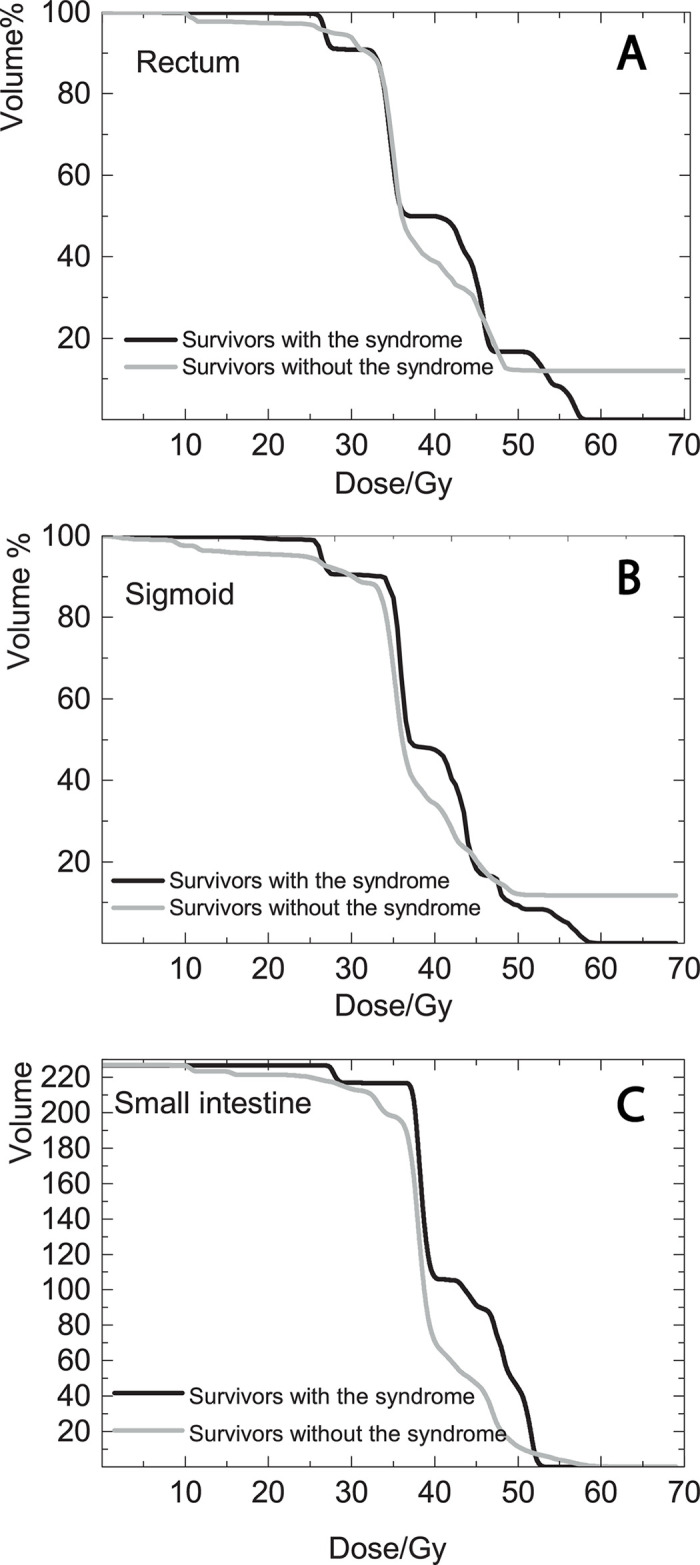
Dose-volume histograms for the survivors with and without ‘excessive mucus discharge’. Mean cumulative DVHs of rectum, sigmoid colon and small intestines for survivors with (black line) and without (grey line) ‘excessive mucus discharge’.

Table 2 presents the mean and max doses in the three OARs for survivors with and without ‘mucus syndrome’. The p-values for the mean doses to the sigmoid and the small intestine were found to be significant. Fig 2 illustrates the mean cumulative DVHs for the survivors with and without ‘mucus syndrome’ for the three OARs. For the rectum, the DVHs for survivors having and not having ‘mucus syndrome’ were well separated for the dose interval 36.0 Gy-46.0 Gy. The DVHs cross at 10.5 Gy, 27.0 Gy, 36.0 Gy, 46.0 Gy, 48.0 Gy and 52.5 Gy. For higher doses, the mean DVH for survivors without ‘mucus syndrome’ was higher than the one for survivors without. For the sigmoid colon, the mean DVHs are well separated for low to intermediate doses (0–27.0 Gy and 30.0–44.0 Gy) and the mean DVH for survivors with ‘mucus syndrome’ was higher than the one for survivors without the syndrome. However, the DVHs cross at 27, 30.0, 44.5, 47.0 and 48.0 Gy. After 48.0 Gy, the mean DVH for survivors without ‘mucus syndrome’ was higher than the one for survivors with the syndrome. For the small intestine, the mean DVH for survivors with ‘mucus syndrome’ was higher than the one for survivors without the syndrome for the dose range 10.5 Gy- 52.0 Gy. The DVHs cross at 52.0 Gy and they merge for doses higher than 59 Gy.

**Table 2:** Mean and maximum doses in the three OARs, for patients with and without ‘excessive mucus discharge’ and correlation coefficients between mean and maximum absorbed doses.

**Survivors with or without ‘excessive mucus discharge’**	**Rectum**	**Sigmoid**	**Small intestines**
	Mean dose (SD)		
**With**	41.7 (9.3)	42.0 (8.8)	41.2 (8.5)
**Without**	38.8 (7.8)	36.8 (7.1)	36.5 (6.9)
p*	0.24	0.022	0.046
	Maximum dose (SD)		
**With**	44.0 (9.4)	45.2 (9.6)	43.4 (8.8)
**Without**	41.3 (8.5)	42.0 (8.3)	41.8 (8.4)
***p***	0.30	0.23	0.57
**Relationship of mean and maximum dose**			
**Pearson correlation coefficient r**	0.97	0.79	0.67

* *p* values from t-test of mean values.

**Fig 3:**
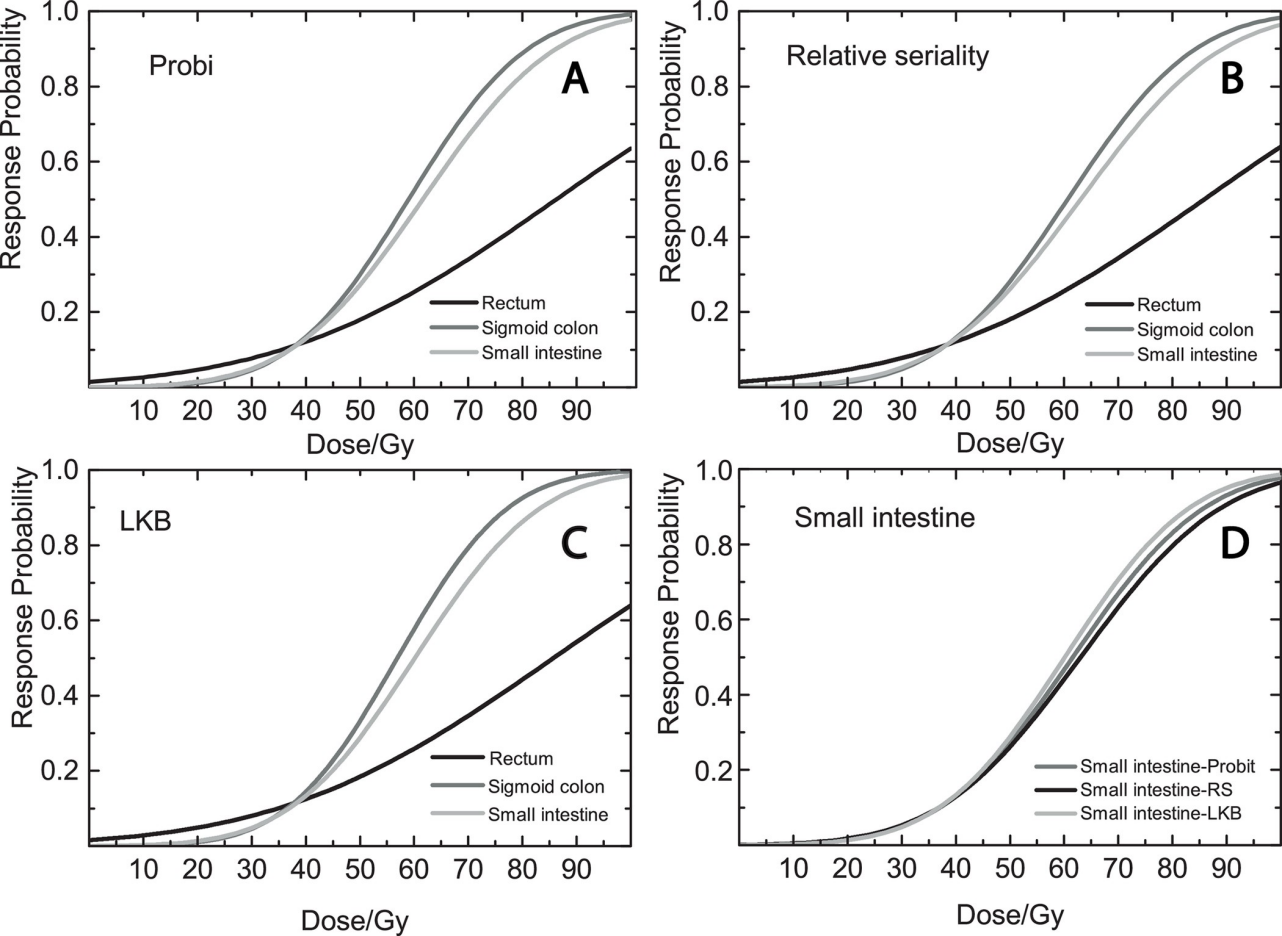
Dose-response curves. The figure illustrates the dose-response curves of mean dose to the: (a) rectum, (b) sigmoid colon and (c) small intestine using the Probit model, the Relative Seriality (RS) model, the LKB model. Panel d illustrates the mean dose to the small intestine for the Probit, Relative Seriality (RS) and LKB models.

Table 3 presents the dose-volume response parameters for the rectum, the sigmoid colon and the small intestine and ‘mucus syndrome’ fitting the data in the Probit, RS and LKB model. The value of *γ*_*50*_ was high for both the sigmoid colon (1.37, 1.31, 1.42) and the small intestine (1.28, 1.23, 1.32) for Probit, RS and LKB models, while for rectum it was low (0.87). The sigmoid colon (AUC: 0.68) and the small intestine (AUC: 0.65) had also the highest AUC value. AIC values were lowest for Probit model from all three OARs but the differences in AIC values between the models were small, which means that there are no large differences in model fitting. Fig 3 illustrates the estimated dose-response relationships. Panels a, b and c show that the sigmoid colon and small intestines have steep dose-response curves for all studied models while the rectum has a shallow curve. Panel d shows that for the small intestine the fitting to the different models was the same for low to intermediate doses but was slightly different for doses above 50 Gy.

**Table 3:** The maximum likelihood estimates of the dose-volume response parameters for the three OAR using Probit, RS, LKB and EUD models and the values for the AUC (area under the ROC curve).

	Rectum	Sigmoid	Small intestines
**Probit**			
**LL**	-35.7	-33.7	-32.1
***D***_***50***_ **(CI)**	86.3 (74.6–102.9)	59.0 (55.1–63.8)	61.6 (56.9–67.4)
***γ***_***50***_ **(CI)**	0.87 (0.75–0.99)	1.37 (1.17–1.57)	1.28 (1.10–1.46)
***AIC***	75.4	71.3	68.2
**Relative Seriality**			
***LL***	-35.7	-33.8	-32.2
***D***_***50***_ **(CI)**	85.9 (74.3–102.2)	60.8 (56.4–65.8)	63.1 (58.0–69.3)
***γ***_***50***_ **(CI)**	0.87 (0.75–0.99)	1.31 (1.12–1.51)	1.23 (1.06–1.41)
***s* (CI)**	2e-07 (1e-100-18.3)	3e-09 (1e-100-0.8)	1e-07 (1e-100- 11)
***AIC***	77.4	73.6	70.4
**LKB**			
**LL**	-35.7	-33.2	-31.8
***D***_***50***_ **(CI)**	85.8 (74.2–100.0)	56.9 (53.4–61.2)	60.2 (55.8–65.5)
***γ***_***50***_ **(CI)**	0.87 (0.75–0.99)	1.42 (1.22–1.64)	1.32 (1.13–1.50)
***m*(CI)**	0.65 (0.57–0.75)	0.39 (0.34–0.46)	0.43 (0.38–0.50)
***n* (CI)**	1.23 (0.009–1.4e+06)	1e+08 (1e+20-1e+20)	1e+08 (1e+20-1e+20)
***AIC***	77.4	72.3	69.6
**EUD**			
***a (1/n)***	0.81 (7.33e-07 111.45)	1e-08 (1.0e-20-1.0e-20)	9.56e-09 (1.0e-20-1.0e-20)
**AUC (CI)**	0.53	0.68	0.65

LL: Log Likelihood value, *D*_*50*_: Dose giving 50% of complication probability (Gy), *γ*_*50*_: Normalized maximum gradient of the dose–response curve, *s*: Relative seriality parameter, *m*: steepness of the dose-response curve, *n*: volume parameter, *a*: volume parameter, AIC: Akaike Information Criterion

## Discussion

Our results indicate that there is a dose-response relationship for the small intestine and the ‘mucus syndrome’. The mean dose to the small intestine correlates with the development of the mucus syndrome. The *γ*_*50*_ values show that the small intestine has steep dose-response curves and thus a strong dose-response relationship (Table 3, Fig 3). The dose-volume response parameters for Probit, RS and LKB models indicate that there is no preferable model to fit these data.

The small intestine was found to have steep dose-response curves *γ*_*50*_ (Probit: 1.28, R.S: 1.23, LKB: 1.32/ m:0.43) and the AUC (0.65) value indicates that the mean doses to the small intestine for survivors with and without the syndrome were fairly well separated. The r (0.67) value suggests that there is linear correlation between the mean and max doses d and thus it is not possible to accurately calculate volume parameters. The estimated volume parameters s, n and a show that the small intestine has a parallel behaviour, but the very wide confidence intervals introduce a significant uncertainty to these values. The p value mean dose to the small intestine were statistically significant. The mean cumulative DVHs for survivors with and without mucus syndrome were also well separated for low to intermediate doses. These observations agree with the results from our NTCP model estimations, which indicates that there is a dose-response relationship between the mean dose to the small intestine and mucus syndrome.

According to the estimated NTCP parameters, the sigmoid colon also seems to have a dose-response relationship. The sigmoid colon had a high *γ*_*50*_ (Probit: 1.37, R.S: 1.31, LKB: 1.42/ m:0.65) and AUC (0.68) value. In Fig 3 and Table 3 we can also see that D_50_ is 59.0 Gy, 60. 8 Gy and 56.9 Gy for Probit, RS and LKB respectively. We were able to estimate a volume parameter for the sigmoid colon for both RS and LKB models indicating a parallel behaviour for both models (s: 3e-09, n: 1e+08). However, the wide confidence intervals and the high r value suggest uncertainty to these values. The p value for the mean dose to the sigmoid colon was significant. However, the mean DVHs for survivors with and without the syndrome cross at several intervals and they are well separated only in two small intervals. This behaviour of the DVHs creates a significant uncertainty in the dose-response results, perhaps due to confounding factor. We believe that the results of the NTCP estimations for the sigmoid colon may be or is the confounding factor.

There was no dose-volume response relationship found for the rectum and ‘mucus syndrome’. The value of *γ*_*50*_ (0.87) was very low and the AUC value was 0.53, which shows that the model cannot distinguish between survivors with or without ‘mucus syndrome’ (Table 3). That was also supported by the DVHs for the rectum. The r (0.97) value was also very high, indicating that the mean and max doses to the rectum were linearly correlated.

Fitting the data to the different models using different a/b values, we found that the fitting is best when using a/b = 1. Practically, that would mean that mucus syndrome is a late adverse effect of radiation therapy.

The strength of this study is the high-quality data that we use and the epidemiological methods developed in-house in order to document patient reported symptoms [19–22]. There are, however, limitations such as the possible errors and the effects of organ movement since the rectum, sigmoid colon and small intestine are highly mobile organs. Another important uncertainty is that we extracted the dose-volume information for only a part of the small intestine. This fact introduces an uncertainty to the estimated dose-volume response relationships. Moreover, there were a few CT scans where the distance of the slices was 2 cm and thus the delineation was difficult.

Thor et al [23] estimated dose-volume response relations for the anal sphincter and mucus discharge after radiotherapy for prostate cancer. AS dose/volume parameters were individual predictors for *Mucous* in SW and the *Mucous* model in SW suggested a parallel behaviour for AS (a = 0.50). High AS doses were important for *Mucous*. Thor et al., page 4 *Acta Oncol*.: Heemsbergen et al reported from physician-based scorings of morbidity, relationships between dose to the superior parts of the rectum and mucous. They found the best predictions for mucus loss were from the dose in the superior 70% of the anorectal map (p 0.007).

Our study indicates that ‘excessive mucus discharge’ is a disctinct syndrome that we may take into consideration when assessing the quality of life of cancer survivors. The small intestine seems to be the major organ associated with the occurrence of the syndrome and thus the separate delineation of the small intestine would be important and useful in reducing the incidence of the ‘excessive mucus discharge’. Trying to reduce and even eliminate the incidence of ‘excessive mucus discharge’, we would suggest that our dose-response estimations be used in developing procedures to meet this goal.

## Supporting information

S1 File (ZIP)

## References

[1] Lind H, Waldenström A-C, Dunberger G, Al-Abany M, Alevronta E., Johansson K. A. et al. Late symptoms in long-term gynaecological cancer survivors after radiation therapy: A population-based cohort study. Br J Cancer. 2011;105(6). 10.1038/bjc.2011.315 21847122 PMC3171018

[2] Johansson MEV, Sjövall H, Hansson GC. The gastrointestinal mucus system in health and disease. Nature Reviews Gastroenterology and Hepatology. 2013. 10.1038/nrgastro.2013.35 23478383 PMC3758667

[3] Johansson MEV, Hansson GC. Immunological aspects of intestinal mucus and mucins. Nature Reviews Immunology. 2016. 10.1038/nri.2016.88 27498766 PMC6435297

[4] Dunberger G, Lind H, Steineck G, Nyberg T, Al-Abany M et al. Self-reported symptoms of faecal incontinence among long-term gynaecological cancer survivors and population-based controls. Eur J Cancer. 2010; 10.1016/j.ejca.2009.10.023 19926277

[5] Lind H, Waldenstrom AC, Dunberger G et al. Late symptoms in long-term gynaecological cancer survivors after radiation therapy: a population-based cohort study. Br J Cancer [Internet]. 2011/08/19. 2011;105(6):737–45. 10.1038/bjc.2011.315 21847122 PMC3171018

[6] Steineck G, Skokic V, Sjoberg F, Bull C, Alevronta E, Dunberger G et al. Identifying radiation-induced survivorship syndromes affecting bowel health in a cohort of gynecological cancer survivors. PLoS One [Internet]. 2017;12(2):e0171461. 10.1371/journal.pone.0171461 28158314 PMC5291512

[7] Buja A, Eyuboglu N. Remarks on Parallel Analysis. Multivar Behav Res [Internet]. 1992;27(4):509–40. 10.1207/s15327906mbr2704_2 26811132

[8] Zientek LR, Thompson B. Applying the bootstrap to the multivariate case: bootstrap component/factor analysis. Behav Res Methods [Internet]. 2007;39(2):318–25. 10.3758/bf03193163 17695360

[9] Kaiser HF. The Varimax Criterion For Analytic Rotation In Factor Analysis. Psychometrika. 1958;23:187–200.

[10] ICRU. Prescribing, recording, and reporting photon beam therapy. ICRU Report 50,. 1993.

[11] Lind H, Alevronta E, Steineck G, Waldenström AC, Nyberg T, Olsson C et al. Defecation into clothing without forewarning and mean radiation dose to bowel and anal-sphincter among gynecological cancer survivors. Acta Oncol [Internet]. 2016;55(11):1285–93. 10.1080/0284186X.2016.1176247 27173757

[12] Jones B, Dale RG, Deehan C et al. The role of biologically effective dose (BED) in clinical oncology. Clin Oncol. 2001; 10.1053/clon.2001.9221 11373882

[13] Philip E. GILL Michael SAUNDERS A., Margaret H. Wright WM. User’s guide for NPSOL 5.0: A FORTRAN package for nonlinear programming. Technical Report SOL 86–6. 2001.

[14] Lyman JT. Complication probability as assessed from dose-volume histograms. Radiat Res Suppl [Internet]. 1985/01/01. 1985;8:S13–9. 3867079

[15] Kallman P, Agren A, Brahme A. Tumour and normal tissue responses to fractionated non-uniform dose delivery. Int J Radiat Biol [Internet]. 1992/08/01. 1992;62(2):249–62. 10.1080/09553009214552071 1355519

[16] Burman C, Kutcher GJ, Emami B, Goitein M et al. Fitting of normal tissue tolerance data to an analytic function. Int J Radiat Oncol Biol Phys [Internet]. 1991;21(1):123–35. 10.1016/0360-3016(91)90172-z 2032883

[17] Reporting Niemierko A. and analyzing dose distributions: a concept of equivalent uniform dose. Med Phys [Internet]. 1997/01/01. 1997;24(1):103–10. 10.1118/1.598024 9029544

[18] Niemierko A. A generalized concept of equivalent uniform dose (EUD). Med Phys. 1999;26:1100 [a. 10.1118/1.598063 9029544

[19] Steineck G, Hunt H, Adolfsson J. A hierarchical step-model for causation of bias-evaluating cancer treatment with epidemiological methods. Acta Oncol (Madr) [Internet]. 2006/06/09. 2006;45(4):421–9. 10.1080/02841860600649293 16760178

[20] Steineck G, Bergmark K, Henningsohn L, al-Abany, M. Dickman, P. W, Helgason, A. et al. Symptom documentation in cancer survivors as a basis for therapy modifications. Acta Oncol [Internet]. 2002;41(3):244–52. 10.1080/02841860260088782 12195743

[21] Bergmark K, Avall-Lundqvist E, Dickman PW et al.Vaginal changes and sexuality in women with a history of cervical cancer. N Engl J Med [Internet]. 1999;340(18):1383–9. 10.1056/NEJM199905063401802 10228188

[22] Steineck G, Helgesen F, Adolfsson J, Dickman P. W, Johansson J. E, Norlen B. J. et al. Quality of life after radical prostatectomy or watchful waiting. N Engl J Med [Internet]. 2002;347(11):790–6. 10.1056/NEJMoa021483 12226149

[23] Thor M, Olsson CE, Oh JH, Petersen, Alsadius S. E., D. Bentzen L. et al. Relationships between dose to the gastro-intestinal tract and patient-reported symptom domains after radiotherapy for localized prostate cancer. Acta Oncol [Internet]. 2015;54(9):1326–34. 10.3109/0284186X.2015.1063779 26340136 PMC4786008

